# Water-Dispersible Phytosterol Nanoparticles: Preparation, Characterization, and *in vitro* Digestion

**DOI:** 10.3389/fnut.2021.793009

**Published:** 2022-01-14

**Authors:** Ao Li, Aixia Zhu, Di Kong, Chunwei Wang, Shiping Liu, Lan Zhou, Ming Cheng

**Affiliations:** ^1^Hubei Key Laboratory of Natural Products Research and Development, College of Biological and Pharmaceutical Sciences, China Three Gorges University, Yichang, China; ^2^Department of Food Science and Technology, Wuhan Polytechnic University, Wuhan, China; ^3^Hubei Key Laboratory of Animal Nutrition and Feed Science, Wuhan Polytechnic University, Wuhan, China; ^4^Wuhan Livestock and Poultry Feed Engineering Technology Research Center, Wuhan Polytechnic University, Wuhan, China

**Keywords:** phytosterol nanoparticles, particle size, encapsulation efficiency, stability, bioaccessibility

## Abstract

For improving solubility and bioaccessibility of phytosterols (PS), phytosterol nanoparticles (PNPs) were prepared by emulsification–evaporation combined high-pressure homogenization method. The organic phase was formed with the dissolved PS and soybean lecithin (SL) in anhydrous ethanol, then mixed with soy protein isolate (SPI) solution, and homogenized into nanoparticles, followed by the evaporation of ethanol. The optimum fabrication conditions were determined as PS (1%, w/v): SL of 1:4, SPI content of 0.75% (w/v), and ethanol volume of 16 ml. PNPs were characterized to have average particle size 93.35 nm, polydispersity index (PDI) 0.179, zeta potential −29.3 mV, and encapsulation efficiency (EE) 97.3%. The impact of temperature, pH, and ionic strength on the stability of fabricated PNPs was determined. After 3-h *in vitro* digestion, the bioaccessibility of PS in nanoparticles reached 70.8%, significantly higher than the 18.2% of raw PS. Upon freeze-drying, the particle size of PNPs increased to 199.1 nm, resulting in a bimodal distribution. The solubility of PS in water could reach up to 2.122 mg/ml, ~155 times higher than that of raw PS. Therefore, this study contributes to the development of functional PS-food ingredients.

## Introduction

Phytosterols (PS), a functional lipid with a cholesterol-like structure, are widely distributed in plants; especially, great amounts of PS originate from oil seeds such as soybean and rapeseed oil (1). Due to its chemical structure similar to cholesterol, PS have a crucial influence on cholesterol metabolism in the body, resulting in serum cholesterol-lowering effects. It is recommended that a daily intake of 2 to 3 g PS can effectively reduce low-density lipoprotein (LDL) cholesterol by 5 to 15% (2). Therefore, PS are considered as an alternative natural substance for the treatment and prevention of hyperlipidemia (3), showing great potential to serve as an ingredient for functional food supplements.

Despite these benefits of PS, the poor solubility in both water and oils and a high melting point easy to crystallize have been challenges for its application. The poor intake of PS from dietary is merely 0.3–0.5 g (4), which hardly accomplishes the health benefits. Therefore, additional PS supplementation is essential to the prevention of cardiovascular disease. Huge efforts have been made to increase the solubility and dispersibility of PS in water, which can be summarized by chemical and physical methods.

Chemical modifications are mostly used to improve the solubility by modulating certain groups (double bonds or hydroxyl groups) of PS. The access to unsaturated fatty acids on the active hydroxyl groups of PS considerably increases their solubility in oil (nearly 10-fold) (5). Compared with free PS, phytostanol shows higher lipophilicity and better cholesterol-lowering effects, mainly relying on artificial synthesis (6). However, the oil phase is required as a carrier for both phytosterol derivatives since the high amount of oil intake became another risk to health (7). To avoid this problem, He et al. proposed an effective method for the preparation of water-soluble phytosterol derivative (phytosterol polyethylene glycol-1000 succinate), which shows a great potential to reduce cholesterol (8).

Colloidal delivery systems are promising physical methods to enhance the water solubility and oral bioavailability of PS. It is reported that both hydroxypropyl β-cyclodextrin inclusion complexes and cyclodextrins could improve the solubility of PS; this is mainly attributed to the manufacture of amorphous form for the encapsulation of PS (9, 10). Recent researches have suggested that PS could be encapsulated by forming nanoemulsions. The translucid and homogeneous β-sitosterol nanoemulsions were fabricated by ultrasonication. The particle size of the phosphatidylcholine-stabilized nanoemulsions ranged from 80 to 200 nm, whereas it ranged from 70 to 900 nm for modified enzymatically lysophosphatidylcholine emulsions (11). No apparent PS crystals were observed due to the solubilization in medium-chain triglycerides (MCT) (12). In addition, it is suggested that the PS crystallization could be limited by the addition of lecithin and monoacylglycerol on nanoemulsion systems (12). Several researchers explored the utilization of the nanostructured lipid carriers for the encapsulation of PS. The lipid phases in the system consist of liquid oils such as MCT and natural edible oils or can be replaced by organic solvent. The solubility or dispensability of PS in the organic phase may affect the encapsulation efficiency (EE). However, the most used surfactants in nanostructured lipid carriers are small molecule emulsifiers (tweens, sorbitan monostearate, spans), which limited the application in food field (13). As an alternative to cholesterol, PS have also been used in the formation of liposomes (14, 15). Wang and colleagues prepared phytosterol liposomes by high-pressure homogenization. They observed that some of the PS were presented as crystals in the inner lumen of the liposomes, whereas the other PS and phytosterol esters were incorporated into the phospholipid bilayer (15).

In more recent work, natural lipid molecules, biocompatible proteins, and polysaccharides have been used to form food-grade nanoparticles. PS that are stabilized by lecithin, soy protein isolate (SPI), whey protein concentrate (WPC), or sodium caseinate (SC) have been investigated due to their good hydrophilic or hydrophobic properties (7). These compounds could also inhibit the formation of the crystal core. Lecithin is the main raw material for the preparation of liposomes and can conjugate with cholesterol or PS to form stable vesicle structures. It was reported that 300 mg of PS emulsified with lecithin had three times the hypolipidemic effect of 1 g free PS (16). SPI is a widely available natural polymer with excellent emulsification properties. It can incorporate lecithin through hydrophobic interactions and electrostatic interactions (17), improving the stability of the emulsified system. To date, protein or lecthin as a carrier has attached special interests, due to their possible interaction with the encapsulated bioactives.

In this study, we used the emulsification–evaporation combined high-pressure homogenization method to producing food-grade phytosterol nanoparticles. The effects of the ratio between PS and SL, the concentration of SPI, and the ethanol volume were investigated on the properties of PNPs. The stability, redispersion behavior, and morphology of PNPs were characterized. The bioaccessibility of PNPs was determined and compared with the free PS. This study confirmed that the fabricated PNPs with SPI and SL achieve a high EE and excellent bioaccessibility, thus showing great potential as a functional PS-food ingredient.

## Materials and Methods

### Materials

PS that consist of β-sitosterol (41%), stigmasterol (25%), and campesterol (30%) were purchased from Wuhan Yuancheng Cocreation Technology Co., Ltd. (Wuhan, Hubei, China). Mixed PS standards (brassicasterol 13%, campesterol 26%, stigmasterol 7%, and β-sitosterol 53%) were obtained from Larodan Fine Chemicals AB Co., Ltd. (Solna, Sweden); 5α-cholestane (>99%, internal standard) and BSTFA-TMCS (99:1) were purchased from Sigma-Aldrich Corporation (St. Louis, Mo, USA). Soybean lecithin (>70%, SL) was supplied by Shanghai Aladdin Biochemical Technology Co., Ltd (Shanghai, China). SPI (>90%, SPI) was purchased from Henan Jianjiu Industrial Co., Ltd. (Henan, China). Pancreatin (from porcine pancreas, P1750, 4 × USP) and pepsin (from porcine gastric mucosal, P7000, 975 units/mg of protein) were purchased from Sigma-Aldrich Corporation (St. Louis, Mo, USA). Bile salt and other reagents and solvents were of analytical grade.

### Preparation of PNPs

PNPs were prepared according to the methods of Leong and Cao (7, 18), with a slight modification. PS (1 g) and SL with the ratio of 1:1 to 1:5 was dissolved in ethanol (8, 12, 16, 20, and 24 ml) to form an organic phase. SPI (0.25–1.25%, w/v) was dispersed in 100 ml deionized water, with pH adjusted to 7.0, and stored overnight at 4°C to constitute the aqueous phase. Under high-speed mixing (T18, IKA, Germany), the organic phase was injected into the aqueous phase at the speed of 10,000 r/min. To form nanodispersion, the primary dispersion was treated by high-pressure homogenization (AH-2010, ATS, Canada) for 6 passes at 90 MPa. After removing ethanol by rotatory evaporation (R3, Buchi, Switzerland) at 40°C, PNPs were successfully produced by adding the deionized water to the initial volume of the dispersions for further analysis. All the experiments were carried out in triplicate.

### Particle Size and Zeta Potential Determination

The particle size, polydispersity index (PDI), and zeta potential of PNPs were determined by dynamic light scattering (DLS) with the use of Malvern Zetasizer Nano-ZS (Malvern Instruments Co. Ltd., Malvern, Worcestershire, UK) at 25°C. The samples were diluted with distilled water to a suitable concentration for the determination. Each measurement was taken in triplicate.

### Encapsulation Efficiency and Loading Amount

#### Pretreatment of Samples

The solubility of raw PS in water was determined according to the method of Wen, with a slight modification (19). To reach supersaturation, excess amount of raw PS was dissolved in deionized water. After 24 h of equilibration in water bath at 37°C, the supernatant of PS solution was centrifuged at 8,000 g for 20 min, followed by gas chromatography analysis. The solubility of PS can be expressed as the ratio of PS content to water volume. PS raw material was dissolved in anhydrous ethanol to form 1 mg/ml PS solution. Two-hundred microliter of the sample was taken into a 5-ml sample bottle and mixed with 200 μl of cholestane (1 mg/ml), which served as an internal standard. The PS in PNPs were determined by the method described by Wang (15). Fresh prepared PNPs were centrifuged at 8,000 g for 20 min to precipitate any unincorporated PS (7, 15). Four-hundred microliter of the supernatant was taken in a 50-ml tube and mixed with 400 μl of 1 mg/ml 5α-cholestane (internal standard). By adding 10 ml of 1.0 mol/L KOH ethanol solution, the mixtures were then saponified at 80°C for 1 h. The samples were cooled at room temperature and extracted by adding 5 ml of water and 10 ml of n-hexane. The organic phase was washed with deionized water for three times and then collected.

The organic solutions were dried under a nitrogen stream. This process has continued to add 100 μl pyridine and 100 μl BSTFA: TMCS (99:1), sealed, and derivatized at a constant temperature at 70°C for 1 h. The samples after reaction were dissolved by 1 ml n-hexane and passed through a 0.22 μm membrane.

#### Gas Chromatography Analysis

Quantitative analysis of PS was determined by GC 7890A, coupled with a flame ionization detector (GC 7890A, Agilent USA). An HP-5 capillary column (30 m × 0.32 mm × 0.25 μm) was used to separate PS. The detections were performed at a constant flow rate (1 ml/min), inlet temperature (300°C), and detector temperature (310°C). The column temperature was kept at 160°C for 2 min, increased with 10°C/min to 280°C and held for 10 min, and finally increased to 300°C at 5°C/min and held for 5 min. The sample was injected with a split ratio of 10:1.

#### Calculation

The EE and loading capacity (LA) of PS in PNPs were calculated by the following equations:


$$\begin{array}{r} EE=\frac{W_{encapsulated}}{W_{total}}\times 100\% \end{array}$$

$$\begin{array}{r} LA=\frac{W_{encapsulated}}{W_{PNPs}}\times 100\% \end{array}$$

where *W*_encapsulated_ was the amount of PS encapsulated in the PNPs; *W*_total_ was the total PS added into the PNPs originally, and *W*_PNPs_ was the weight of the PNPs.

### Stability of PNPs

The storage stability of PNPs prepared at optimal condition was evaluated using a Turbiscan Lab (Formulaction, France). The samples prepared on days 0, 7, and 14 were transferred to a cylindrical glass cell and then scanned every 1 min for 60 min at 25°C, and the change in backscattered light intensity in unit time was taken as a measure of the stability (20).

### Effect of pH, Temperature, and Ionic Strength on PNPs

The stability of the PNPs in different environments (pH, temperature, and concentration of ionic strength) was evaluated according to the particle size and zeta potential determined by DLS. Each determination was performed in triplicate.

#### pH Stability

To understand the response toward different pH, a series of samples with aqueous phases adjusted to values ranging from pH 2–7 using either 0.1 M NaOH or 0.1 M HCl solution.

#### Temperature Stability

The influence of temperature ranging from 4°C, 25°C, 37°C, and 60°C on PNPs stability was examined to study the thermal stability.

#### Salt Stability

To investigate the stability of PNPs under the condition of salts stress, the effect of ionic strength on stability of PNPs was examined by dissolving in different concentrations of either NaCl (0, 10, 25, 50, 100, 150, and 200 mmol/L) or CaCl_2_ (0, 10, 25, 50, 100, 150, and 200 mmol/L).

### Morphology Observation

#### Scanning Electron Microscope

The morphology of raw PS was evaluated with SEM (S-3000N, Hitachi, Japan). A small amount of the powder was fixed to a stainless-steel stage with double-sided tape and then sprayed with gold using an E-1045 particle sputterer. The samples were placed in a chamber of SEM for observation.

#### Transmission Electron Microscopy

The morphology of PNPs was observed with TEM (JEM-2100, JEOL Ltd. Japan). The prepared sample was diluted to a phospholipid concentration of 0.8 mg/ml with deionized water and then dropped onto a special copper grid. The excess liquid was blotted up on filter paper. Finally, the sample was negatively stained with 3% phosphotungstic acid and dried by natural evaporation and then for TEM observation.

### X-Ray Diffractometry

The freeze-dried samples were obtained by Lyophilizer (FD-50, Christ company, Germany) and then were evaluated by XRD (Empyrean, Malvern Panalytical, the Netherland) by placing them on the XRD glass slides. The operation conditions were at 40 kV and 40 mA with Cu as the X-ray radiation and then scanned from 3° to 40° at a speed of 10° per min with a gap of 0.02°.

### Differential Scanning Calorimetry

The freeze-dried samples were thermal analyzed using a DSC (Q2000, TA Instruments, CA, USA). The measurements were taken in the range of 25°C−200°C using an aluminum crucible as a reference. The sample heating rate was set to 10 °C/min with N_2_ flow rate at 20 ml/min.

### Fourier Transform Infrared Spectrometer

The FTIR (Nexus 670, Thermo Nicolet, USA) spectra of raw materials, physical mixture of the complexes, and freeze-dried PNPs were analyzed at 25°C, using the KBr pellet technique. The samples were detected in the region of 400–4000 cm^−1^, with 2 cm^−1^ of resolution.

### *In vitro* Bioaccessibility

The *in vitro* bioaccessibility of free PS and PNPs was evaluated with simulated gastric and intestinal digestive fluid, modified with reference to the reports of Cao et al. (7) and Liu et al. (21). The simulated gastric fluid (SGF) was prepared by dissolving 2 g NaCl in 1 L of deionized water, adjusting the pH to 2.0 with 0.1 mol/L HCl, and storing at 4°C. The simulated intestinal fluid (SIF) was formed with 6.8 g KH_2_PO_4_ in 1 L of deionized water, adjusting the pH to 7.0 with 0.1 mol/L NaOH. Before digestion, pepsin and trypsin were dispersed in SGF and SIF, respectively. The supernatant was extracted by centrifugation at 3,000 r/min.

Fresh PNPs (10 ml) were mixed with 9 ml of SGF in a 50-ml centrifuge tube and adjusted the pH to 2.0. The mixture was preincubated at a constant temperature water bath at 37°C and shook at 95 r/min for 10 min. One milliliter of pepsin (80 mg/ml) was then added to start the gastric digestion (0–60 min). Ten milliliter of digestion solution was removed after 60 min and mixed with 9 ml of SIF. The pH was rapidly adjusted to 7.0 with 4 mol/L NaOH, followed by the addition of 160 mg of bile salts. After 10 min preincubated at water bath shaker, 1 ml of trypsin (160 mg/ml) was added to the final solution and shook for 120 min. For comparison, the raw PS was dissolved in ethanol with the same concentration as PNPs and then subjected to the same digestion process. All of the experiments were conducted in triplicate with data reported as the mean ± standard deviation.

The percent bioaccessibility of PS was calculated as follows:


$$\begin{array}{r} Bioaccessibility\;\left(\%\right)=\frac{W_{soluble}}{W_{total}}\times 100\% \end{array}$$

where the mass of solubilized PS (*W*_*soluble*_) was the product in aqueous phase after *in vitro* lipolysis, it is determined by the PS in supernatant after 8,000 g centrifugation for 20 min to remove any insoluble in the dispersions.

## Results and Discussion

### Preparation of PNPs

#### Effect of the Ratio of PS to Soybean Lecithin on the Properties of PNPs

**Figure 1:**
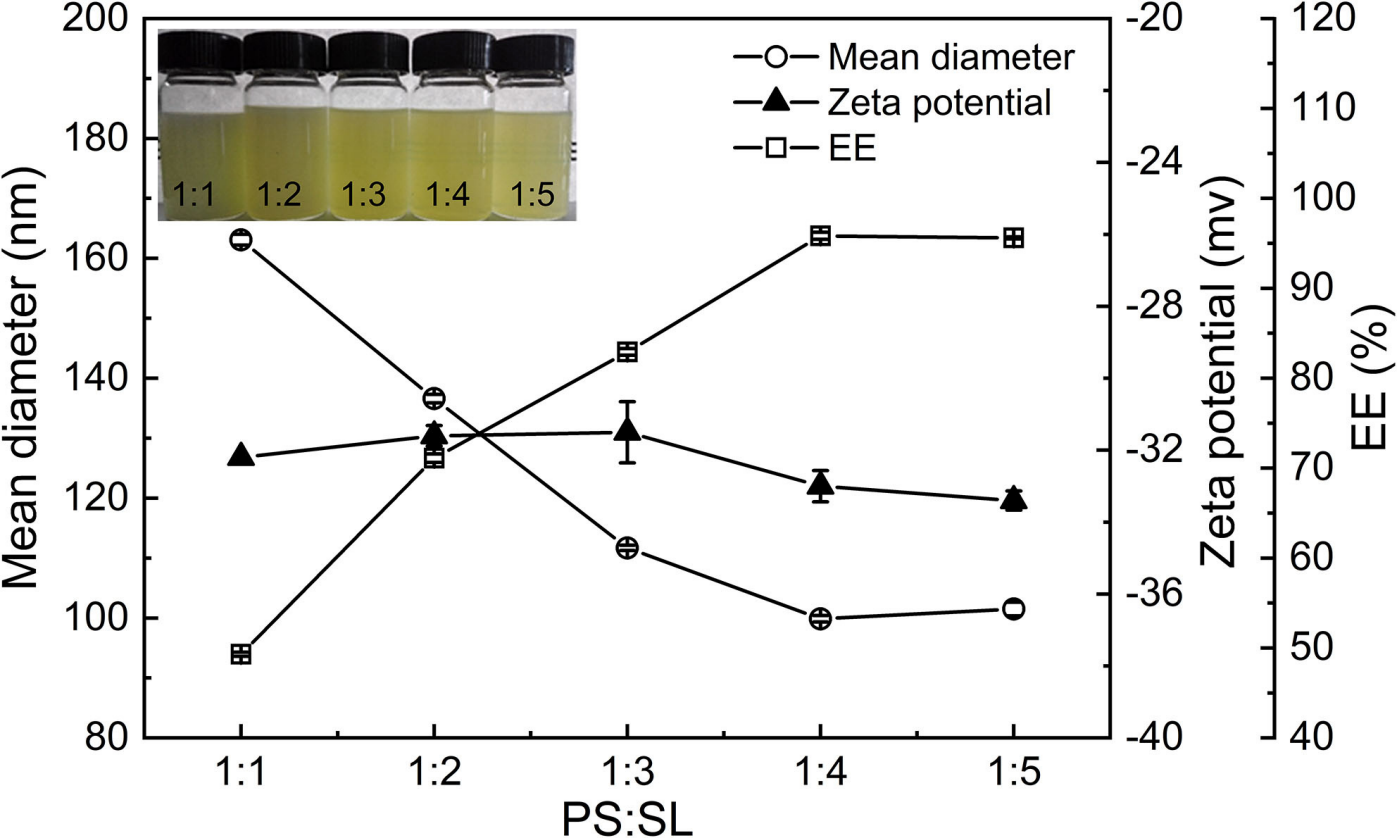
Effect of the ratio of PS:SL on the particle size, zeta potential, and EE of PNPs.

Figure 1 shows the particle size, zeta potential, and EE of PNPs prepared by various PS to SL ratios in 12 ml of ethanol, SPI concentration 1% (w/v), and homogenized at 900 bar for six passes. The PNPs samples were gradually clarified as the SL ratio increased. The particle size of the PS nanoparticles significantly decreased from 163.1 to 99.9 nm with the ratio of PS to SL increasing. A slight increase of size was observed when further increasing the ratio to 1:5. The zeta potential was around −32 mV, which indicates excellent stability of PNPs. Besides the particle size and zeta potential, the EE of PS is an important parameter to be considered. The EE gradually increased up to 1.9-fold (from 50 to 95%), as the ratio increased from 1:1 to 1:4. At the low ratio, PS cannot be fully combined with the phospholipid and therefore tend to precipitate in the solution. Continuing to increase the proportion of SL to 1:5, the EE of PNPs does not significantly improve, but LA will decrease due to the increased weight of SL. Thus, considering the particle size and EE, the optimal PS to SL mass ratio was achieved at 1:4.

#### Effect of the Concentration of SPI on the Properties of PNPs

**Figure 2:**
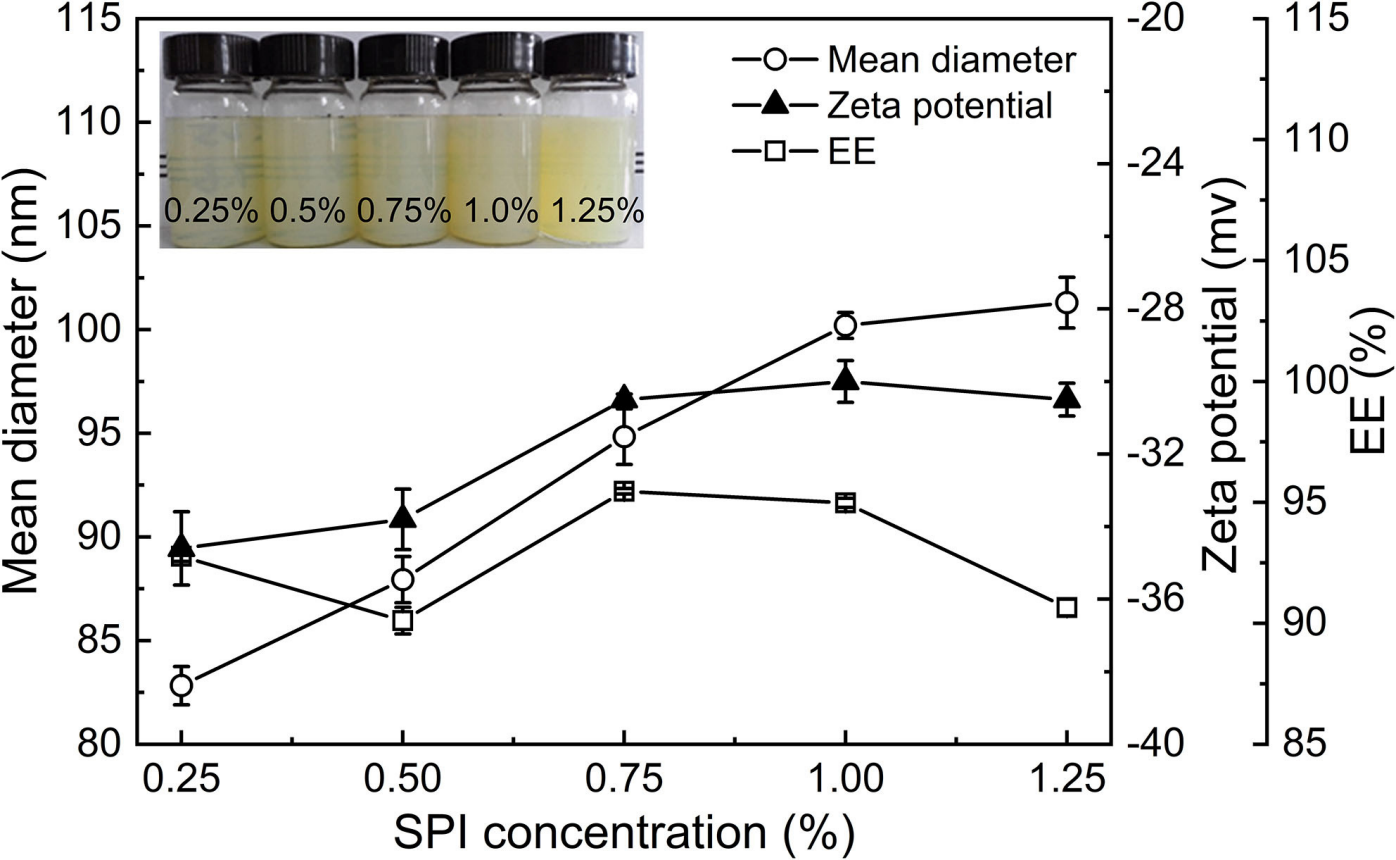
Effect of the SPI concentration on the particle size, zeta potential, and EE of PNPs.

The influence of concentration of SPI on particle size, zeta potential, and EE of PNPs were investigated, as shown in Figure 2. Due to the addition of a large molecule of protein, the particle size of PNPs was increased from 82.8 to 101.3 nm with the increase of SPI content from 0.25 to 1.25%, and the products were gradually cloudy (Figure 2). The combination of SPI and SL through hydrophobic and electrostatic interactions affects the structure of the nanodispersion, thus improving the physical stability of the system. The interactions between SPI and SL were in accordance with the previous study (22). It can be seen in Figure 2 that the zeta potential increased from −34.6 mV to −30 mV, and the addition of SPI did not have a significant effect on the EE of PS. The observation suggested that the SPI content of 0.75% was the optimal concentration to form stable PNPs.

#### Effect of the Volume of Ethanol on the Properties of PNPs

**Figure 3:**
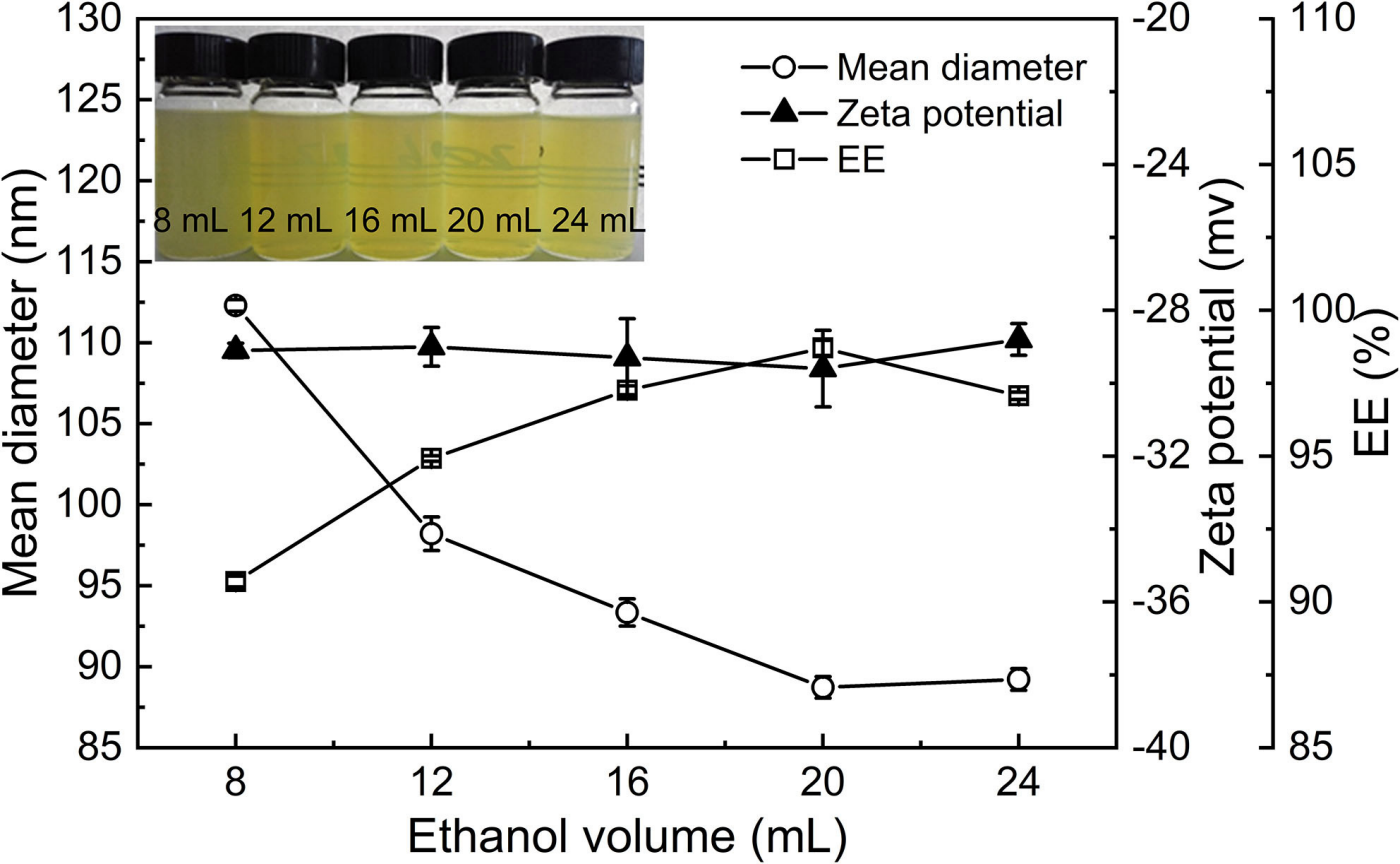
Effect of the ethanol volume on the particle size, zeta potential, and EE of PNPs.

Figure 3 displayed the particle size, zeta potential, and EE of PS nanoparticles as affected by the volume of ethanol. It was observed that the volume of ethanol has few impacts on the zeta potential of PNPs, which remained around −29 mV. When the volume of ethanol increased from 8 to 20 ml, the particle size of PNPs considerably decreased from 112.3 to 89.2 nm, and the EE progressively increased from 90.7 to 98.7% (Figure 3). This result confirmed the importance of ethanol as a cosolvent, which facilitates the dispersion of PS in nanodispersion. The contribution of adequate phytosterol–lecithin combination resulted in smaller particle size and higher EE. Additionally, when the volume of ethanol was further increased to 24 ml, ethanol exposed the hydrophobic groups of proteins, thus leading to the aggregation. One point is noteworthy that, when the volume of ethanol is higher than 16 ml, many bubbles were generated and affected the formation of nanodispersion. Therefore, the addition of 16 ml of ethanol would be appropriate in our condition.

### Stability of PNPs

#### Storage Stability

To investigate the storage stability of PNPs, Turbiscan stability index (TSI) of fresh PNPs solution was obtained by monitoring the evolution of the backscattered and/or transmitted light intensities for fresh PNPs and PNPs stored for 7 days, as shown in Supplementary Figure S1. The TSI of fresh PNPs tends toward 0.04, the results of TSI stabled at 0.25 for PNPs stored for 7 days, and no crystallization of PS was observed in nanodispersion systems, which indicate that the nanoparticles were relatively stable. Due to the disruption of PNPs dispersion systems by mold, the TSI of PNPs stored for 14 days was significantly increased to 1.2 (date is not shown).

#### Effect of pH, Temperature, and Ionic Strength on the Stability of PNPs

**Figure 4:**
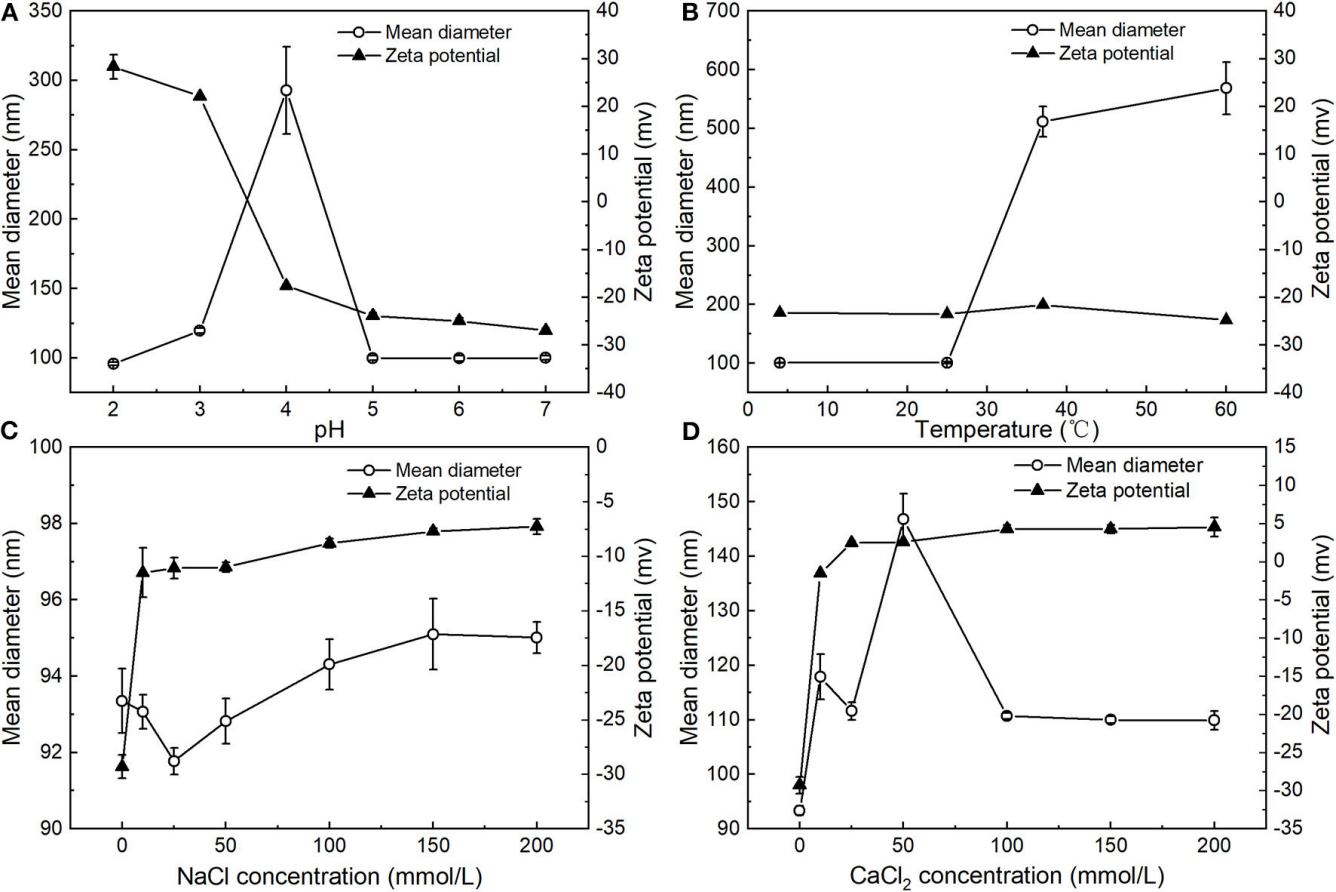
Influence of pH **(A)**, temperature **(B)**, concentration of NaCl **(C)**, and CaCl_2_ **(D)** on mean particle diameter and zeta potential of PNPs.

The influence of pH on the particle size and zeta potential of the PNPs was investigated (Figure 4A). Overall, the zeta potential of the PNPs converts from positive at low pH to negative at high pH, with a point of zero charges between pH 3 and 4. This phenomenon is similar to the typical protein-coated emulsions (23, 24). At the low pH below the pI of SPI, the amino groups are positively charged (${-\text{NH}}_{3}^{+}$), and the carboxyl groups are neutral (-COOH). Thus, the zeta potential of PNPs represented a net positive charge. PNPs have zero charges around pH 3.5, which is slightly lower than the isoelectric point (pI, around 4.5) of SPI. This result may be due to differences in the conformation of a globular protein when interacting with PS compared to in an aqueous solution, indicating the likely unfold of the SPI at the interface and the expose of positive groups. Whereas, at high pH>pI, the zeta potential of PNPs represented a net negative charge, the mean particle size of PNPs remained relatively small (around 95 nm) except from pH 4 around the isoelectric point, which is consistence with the low net droplet charge at pH 4. The droplets tend to aggregate due to the relatively weak electrostatic repulsion. Tokle also indicated that the lactoferrin emulsion underwent the similar trend variation (25).

Figure 4B showed that the particle size was nearly unaffected at 4°C and 25°C for 24 h and remained below 100 nm, demonstrating the excellent stability of PNPs. Unlike the PS nanoemulsions, nanoemulsions displayed a significant increase of particle size at 4°C (11), whereas obvious droplet aggregation was observed when stored at 37°C and 60°C. Additionally, the droplet size was largely increased to 511.5 ± 25.6 nm at 37°C, 568.6 ± 44.5 nm at 60°C, respectively. There was no obvious change in zeta potential of the PNPs in different temperatures.

The zeta potential and particle size of the PNPs in the presence of various NaCl and CaCl_2_ concentrations were measured. For the addition of NaCl, the mean particle size of PNPs was around 95 nm ranging from 10 to 200 mmol/L NaCl (Figure 4C). There was no observed phase separation or any significant change in the droplet size, suggesting the good stability of salt-induced aggregation. In addition, the zeta potential of the system was decreased with the increase of NaCl concentration. Since the addition of NaCl leads to the neutralization of the negative charge of soybean isolated protein, which increases the zeta potential of nanoparticles, the stabilization of the PNPs is mainly through the hydrophobic interaction and electrostatic interaction between SPI and SL.

Regarding the addition of CaCl_2_, the particle size of PNPs was slightly increased to 117.9 ± 4.1 nm after adding 10 mmol/L CaCl_2_ (Figure 4D), since Ca^2+^ neutralized the negative charge on the surface of the protein. With an increase of CaCl_2_ concentration from 25 to 200 mmol/L, particle size remained unchanged, suggesting that bridging and ion-binding effects did not lead to droplet aggregation, thus destabilizing the system. When increasing the concentration of CaCl_2_ from 10 to 25 mmol/L, the zeta potential of PNPs was converted from negative to positive. The net zeta potential was gradually increased with Ca^2+^ concentration. Therefore, PNPs showed relatively stable property against CaCl_2_-induced aggregation. Increasing ionic strength can easily induce droplet flocculation in typical protein stabilized emulsion (26) and lecithin emulsion (27). In our study, it is observed that the formulation of PS-SL provided good stability.

### Morphology of PNPs

**Figure 5:**
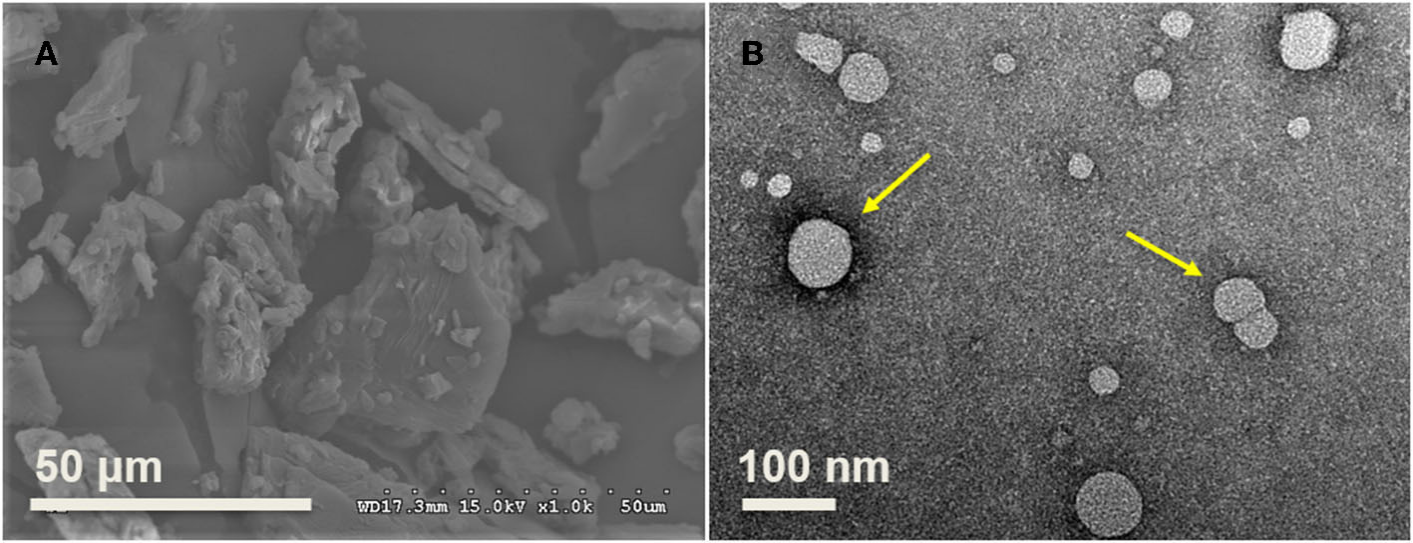
Morphology of raw materials and fresh prepared PNPs. **(A)** PS in the range from 20 to 50 μm; **(B)** Fresh prepared PNPs with size below 80 nm.

The SEM results indicated that the size of raw PS ranges from 20 to 50 μm based on the analysis of ImageJ (Figure 5A), consistent with the previous study, and varied from 20 μm to 115 μm (28). TEM was used to observe the morphology of PNPs prepared at optimal conditions, as shown in Figure 5B. The result indicated that most PS particles were spherical, with a particle size below 80 nm. The TEM result is slightly lower than the particle size analyzed by DLS. The nanoparticles were evaluated in dried form, and the loss of water could induce the decrease of measured particle size. It is consistent with the previous report on gold nanoparticle sizes (29).

### Characterization of Freeze-Dried PNPs

#### Redispersion Behavior

**Figure 6:**
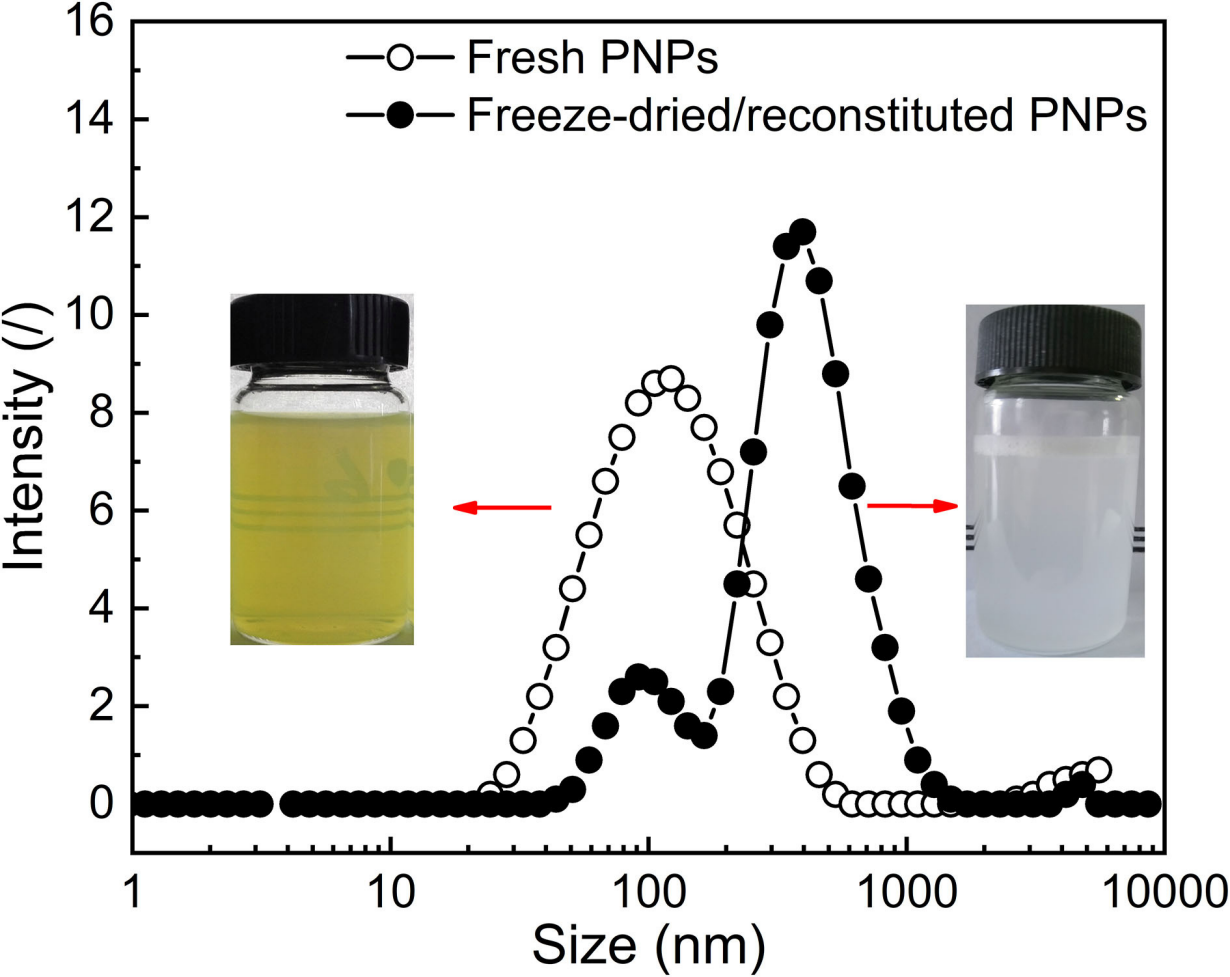
Representative particle size distribution and visual observation of both fresh PNPs solution and freeze-dried or reconstituted dispersions.

The optimal PNPs were formed with the ratio of PS: SL: SPI equal to 1:4:0.75 (w/w/w). To investigate the effect of freeze-drying treatment on the redispersion behavior of PNPs, the visual observation, particle size distribution, zeta potential, and dispersibility of both fresh PNPs solution and freeze-dried or reconstituted dispersions were determined as presented in Supplementary Table S1. Compared with the turbid and light yellow in fresh PNPs solution, freeze-dried or reconstituted dispersions represented milky (Figure 6). This finding was unexpected and suggested that SL might play a role in the color change. It is assumed that the yellow color of fresh PNPs might be contributed to the SL, and part of PS-SL complex (similar to liposome) is not fully encapsulated by SPI. During the drying process, PS-SL complex reassembles with the SPI shell of PNPs and did not separate in the redispersion. Therefore, the size distribution indicated that freeze-drying treatment led to a slight shift toward a more extensive size distribution, corresponding to the increased PDI from 0.18 ± 0.011 to 0.44 ± 0.029. The particle size increased from 93.35 ± 0.84 to 199.1 ± 2.05 nm. The zeta potential of the nanoparticles was almost unaffected after the freeze-drying treatment. The solubility of PS in water could reach up to 2.122 mg/ml, ~155 times higher than that of raw PS. All these results demonstrated that the fabricated PNPs showed good redispersion behavior in water.

#### Encapsulation of PS (DSC, XRD, and FTIR)

**Figure 7:**
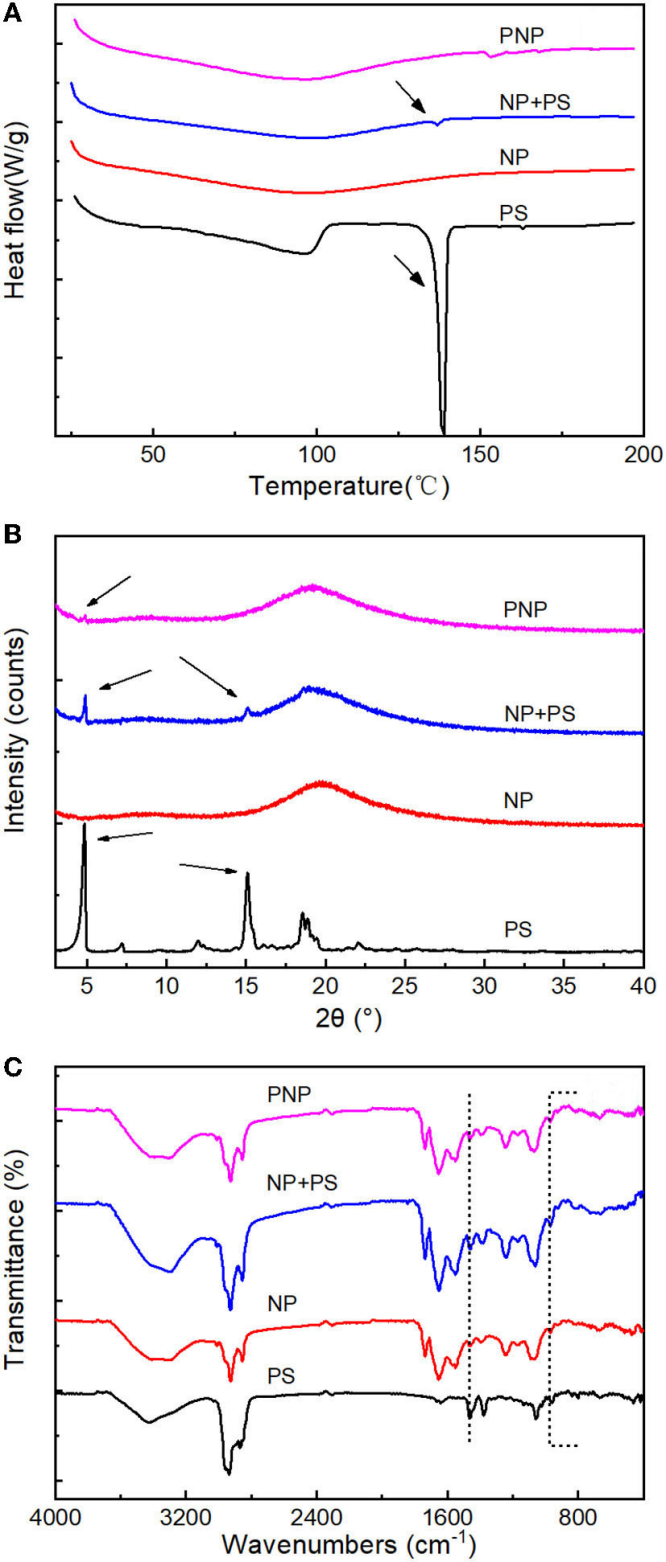
Encapsulation analysis. **(A)** DSC thermograms of PS, nanoparticles (NP), NP+PS and PNP; **(B)** XRD patterns of PS, nanoparticles (NP), NP+PS and PNPs; and **(C)** FTIR spectra of PS, nanoparticles (NP), NP+PS and PNPs.

To further investigate the encapsulation of freeze-dried PS in PNPs, formed at optimal conditions, DSC, XRD, and FTIR were performed, as shown in Figure 7.

Free PS exhibited an endothermic peak with temperatures at 140°C, compared with the small peak which was observed in the mixture of free PS with NP. The prominent endothermic peak was not detectable for the PNPs, which is similar to the empty NP (Figure 7A), indicating excellent encapsulation of PS in the nanoparticles. The free PS displayed firm characteristic peaks at 5° and 15°. The simple physical mixture of free PS with NP showed the same XRD pattern of PS. In contrast, no diffraction lines were observed for the PNPs (Figure 7B), and it could be attributed to the addition of SL that prevented the PS from crystallization. The encapsulation of PS can be further confirmed by FTIR. As shown in Figure 7C, PS have an absorption peak at 3,428 cm^−1^ due to the stretching vibration of the hydroxyl group; the same term was found at 3,000 to 2,800 cm^−1^ (C-H) and 1,056 cm^−1^ (C-O-C). Compared with blank NP (without PS), the difference occurs at 968 cm^−1^, 960 cm^−1^ for stretching of trans-disubstituted olefins (=C-H) and 836 cm^−1^, 802 cm^−1^ for trisubstituted olefins (=C-H). The transmittances were observed to decrease compared with the physical mixture of NP and PS, almost negligible in PNPs. The same tendency could be found at 1,458 cm^−1^ and 1,381 cm^−1^ attributed to saturated C-H. It demonstrated the good encapsulation of PS in PNPs.

### *In vitro* Bioaccessibility of PS

*In vitro* digestion model consisting of sequential gastric (60 min) and intestinal (120 min) digestion was utilized to evaluate the bioaccessibility of free PS or encapsulated PS in the nanoparticles. Under the action of digestive enzymes, the bioaccessibility of free PS was merely 18.23%. For the encapsulated PS in the nanoparticles, the bioaccessibility reached 70.80%. This improvement might be largely attributed to the unique structure of PNPs, and the nanoscale structure significantly decreased the PS particle size and crystallization. PS can readily be dissolved into the gastric and intestinal environment and then incorporated into bile micelle structures.

After encapsulated, the water-dispersible PS was fabricated on nanoscale as described in Table 1. The PNPs prepared with SPI and SL showed good solubility in water and reached 2.122 mg/ml, just below the complex formed with β-cyclodextrin (8.690 mg/ml), and Meng demonstrated that the unique structure of cyclodextrin/PS inclusion complex with central cavity provided a lipophilic microenvironment for the encapsulation of PS (10). It was worth mentioning that the LA of fresh PNPs could reach 171.67 mg/g, which is at the same level with zein-based (32) nanodispersion and SPI-based nanodispersion (34). The LA of PS nanoemulsion prepared by self-microemulsion was 87.22 mg/g (30), which is lower than the highest LA (273 mg/g) in SC-based PS nanoparticles (7). Cao also confirmed that SC was the most effective emulsifier for the PS nanoformulation (7). However, the small amount of SC applied in the preparation resulted in a relatively lower EE of 64% (7).

**Table 1:** Recent researches on water-dispersible phytosterol delivery systems.

**Systems**	**Materials**	**Size (nm)**	**LA (mg/g)**	**Solubility/** **dispersibility (mg/ml)**	**Bioaccessibility (%)**	**Reference**
Complex	β-cyclodextrin	/	/	8.690	/	(10)
Nanoemulsion	Tween 60, PEG 400	48.85	87.22	/	/	(30)
Nanodispersion	Sucrose fatty acid esters	2.8–259.9	/	0.2304–0.5046	/	(31)
Nanodispersion	Zein	336.74	194	/	/	(32)
Nanodispersion	Pectin, zein	584.4	154.5	/	/	(33)
Nanodispersion	SPI	97.2	191	/	/	(34)
Nanoparticle	Nanoporous starch aerogels	70	195	/	27.7	(1)
Nanoparticle	Nanoporous starch aerogels	/	/	/	16.8/29.2	(28)
Nanoparticle	Nanoporous starch aerogels	59–87	99	/	/	(35)
Nanoparticle	SPI/WPC/SC	122.5–204.5	273	/	29.2	(7)
Nanoparticle	SPI, SL	93.35	171.67	2.122	70.8	This study

In other colloidal systems (Table 1), the nanoparticle prepared with nanoporous starch aerogels showed a PS bioaccessibility of 27.7% (1). The protein-based nanoparticle formed with SC also indicated an increase of bioaccessibility up to 29.2% (7). Due to the hydrolysis of protein and starch in the gastrointestinal environment, bioaccessibility is relatively low. In our condition, the fresh PNPs produced by SPI and SL achieved the highest bioaccessibility (70.8%). Therefore, it could conceivably be hypothesized that SL might play an essential role in enhancing the bioaccessibility of PS.

## Conclusion

In this study, PS nanoparticles with SPI and SL were successfully fabricated by emulsification–evaporation combined high-pressure homogenization method. The optimum conditions were selected at PS (1%, w/v): SL ratio of 1:4, SPI content of 0.75% (w/v), and ethanol volume of 16 ml. The nanoscaled PNPs achieved high EE of PS and displayed moderate stability toward temperature, pH, and ionic strength. The freeze-dried PNPs exhibited good redispersion behavior in water. The results suggest an effective approach for the development of food-grade PNPs with good water dispersibility. Compared with the raw PS, the incorporation of SL with PS in PNPs showed remarkable improvement of bioaccessibility. Despite these promising results, *in vivo* experiments on animals should be considered to confirm the enhanced bioaccessibility of PNPs.

## Data Availability Statement

The original contributions presented in the study are included in the article/Supplementary Material, further inquiries can be directed to the corresponding authors.

## Author Contributions

MC, CW, and AL involved in conceptualization. SL involved in analysis. MC, DK, and AL performed methodology. MC conducted the investigation. MC and AL wrote the original draft. MC, SL, and LZ involved in writing, reviewing, and editing. AZ and CW performed funding acquisition. CW and MC supervised the study. All authors contributed to the article and approved the submitted version.

## Funding

This research was funded by the National Key Research and Development Project of China (2017YFD0500603) and the Open Project of Hubei Key Laboratory of Animal Nutrition and Feed Science (DKXY2020012).

## Conflict of Interest

The authors declare that the research was conducted in the absence of any commercial or financial relationships that could be construed as a potential conflict of interest.

## Publisher's Note

All claims expressed in this article are solely those of the authors and do not necessarily represent those of their affiliated organizations, or those of the publisher, the editors and the reviewers. Any product that may be evaluated in this article, or claim that may be made by its manufacturer, is not guaranteed or endorsed by the publisher.
