# Supplementary material for: Water-Dispersible Phytosterol Nanoparticles: Preparation, Characterization, and in vitro Digestion
Source: Front Nutr. 2022 Jan 14;8:793009. doi: 10.3389/fnut.2021.793009 (PMC8795707; doi:10.3389/fnut.2021.793009)
Supplement: Supplementary file 1 [file Data_Sheet_1.docx]

Fig. S1. TSI values measured in 60 min for fresh PNPs and PNPs stored for 7 days.

Table S1. The particle size, PDI, zeta potential and solubility for both fresh PNPs and freeze-dried/reconstituted PNPs.

| **Samples** | **Size(nm)** | **PDI** | **Zeta potential (mV)** | **Solubility (mg/mL)** |
| --- | --- | --- | --- | --- |
| Fresh PNPs | 93.35±0.84 | 0.18±0.011 | -29.3±1.07 | / |
| Freeze-dried/  reconstituted PNPs | 199.1±2.05 | 0.44±0.029 | -32.8±1.58 | 2.122 |
